# Omicron: A Heavily Mutated SARS-CoV-2 Variant Exhibits Stronger Binding to ACE2 and Potently Escapes Approved COVID-19 Therapeutic Antibodies

**DOI:** 10.3389/fimmu.2021.830527

**Published:** 2022-01-24

**Authors:** Masaud Shah, Hyun Goo Woo

**Affiliations:** ^1^ Department of Physiology, Ajou University School of Medicine, Suwon, South Korea; ^2^ Department of Biomedical Science, Graduate School, Ajou University, Suwon, South Korea

**Keywords:** SARS-CoV-2, Omicron, ACE2, antibodies, immune escape, therapeutic

## Abstract

The new SARS-CoV-2 variant of concern “Omicron” was recently spotted in South Africa and spread quickly around the world due to its enhanced transmissibility. The variant became conspicuous as it harbors more than 30 mutations in the Spike protein with 15 mutations in the receptor-binding domain (RBD) alone, potentially dampening the potency of therapeutic antibodies and enhancing the ACE2 binding. More worrying, Omicron infections have been reported in vaccinees in South Africa and Hong Kong, and that post-vaccination sera poorly neutralize the new variant. Here, we investigated the binding strength of Omicron with ACE2 and monoclonal antibodies that are either approved by the FDA for COVID-19 therapy or undergoing phase III clinical trials. Computational mutagenesis and free energy perturbation could confirm that Omicron RBD binds ACE2 ~2.5 times stronger than prototype SARS-CoV-2. Notably, three substitutions, i.e., T478K, Q493K, and Q498R, significantly contribute to the binding energies and almost doubled the electrostatic potential (ELE) of the RBD^Omic^–ACE2 complex. Omicron also harbors E484A substitution instead of the E484K that helped neutralization escape of Beta, Gamma, and Mu variants. Together, T478K, Q493K, Q498R, and E484A substitutions contribute to a significant drop in the ELE between RBD^Omic^–mAbs, particularly in etesevimab, bamlanivimab, and CT-p59. AZD1061 showed a slight drop in ELE and sotrovimab that binds a conserved epitope on the RBD; therefore, it could be used as a cocktail therapy in Omicron-driven COVID-19. In conclusion, we suggest that the Spike mutations prudently devised by the virus facilitate the receptor binding, weakening the mAbs binding to escape the immune response.

## Introduction

Since its emergence, severe acute respiratory syndrome coronavirus 2 (SARS-CoV-2) has been found to continuously evolve and raise new variants of concerns (VOCs) to avoid host hostilities, i.e., evade the host immune response, increase transmission, and aggress the pathogenesis of coronavirus disease 2019 (COVID-19). This host adaptation by the virus has been demonstrated by the rise of VOCs, including Alpha, Beta, Gamma, and Delta variants that weaken the neutralizing efficacy of antibodies (1–4). Most recently, a new strain of the SARS-CoV-2 named Omicron by the World Health Organization has emerged in South Africa (November 24, 2021) and spread worldwide within a short period. Researchers around the globe are racing to determine whether Omicron poses a threat to the immunity induced by the COVID-19 vaccine (5).

Omicron harbors many novel mutations in structural and non-structural proteins, leading to serious concerns over vaccine failure, immune escape (5), and increased transmissibility. More than 32 mutations were found in the Spike protein alone, where 15 of these mutations reside in the receptor-binding domain (RBD), which are vital to both receptor and viral neutralizing antibodies. The non-structural proteins encoded by the ORF1ab contain mutations in the nsp3 (K38R, V1069I, Δ1265, L1266I, A1892T), nsp4 (T492I), nsp5 (P132H), nsp6 (Δ105-107, A189V), nsp12 (P323L), and nsp14 (I42V). Nsp3 (Plpro) and nsp5 (3Clpro, main protease) are proteases that cleave the polypeptide encoded by ORF1a and ORF1ab. 3Clpro and nsp12 [RNA-dependent RNA-polymerase (RdRp)] are primary targets for drugs that block the polypeptide cleaving and viral protein synthesis (6). Using structural models and as confirmed by the preliminary data in a preprint study (7), we found that mutations in nsp5 and nsp12 are not close to the active site and may not hinder the effect of antiviral drugs; nonetheless, these proteins play a vital role in innate immune response (interferon induction), requiring further experimental investigation (6). Omicron also had mutations in the other structural proteins, including Envelope (E) (T9I), Membrane (M) (D3G, Q19E, and A63T), and Nucleocapsid (N) (P13L, Δ31-33, R203K, G204R), further enhancing their infectivity. Since N protein is highly immunogenic (8, 9), these mutations could help escape the host immune response.

In addition, Omicron had multiple mutations in the Spike protein, which are associated with increased infectivity and antibody evasion. Out of 32 mutations, half of them hold the potential to dampen the potency of therapeutic antibodies and enhance the ACE2 binding. Omicron has also been shown to infect triple-vaccinated individuals who have received BNT162b2 jabs (10). Here, we conducted molecular modeling and mutational analyses to delineate how the new variant enhances its transmissibility and escapes against the FDA-approved Spike-neutralizing COVID-19 therapeutic antibodies. Our results may provide new insights into therapeutic management against the infection caused by Omicron.

## Results

### Mutations in the Omicron RBD Strengthen the Spike–ACE2 Interaction

**Figure 1:**
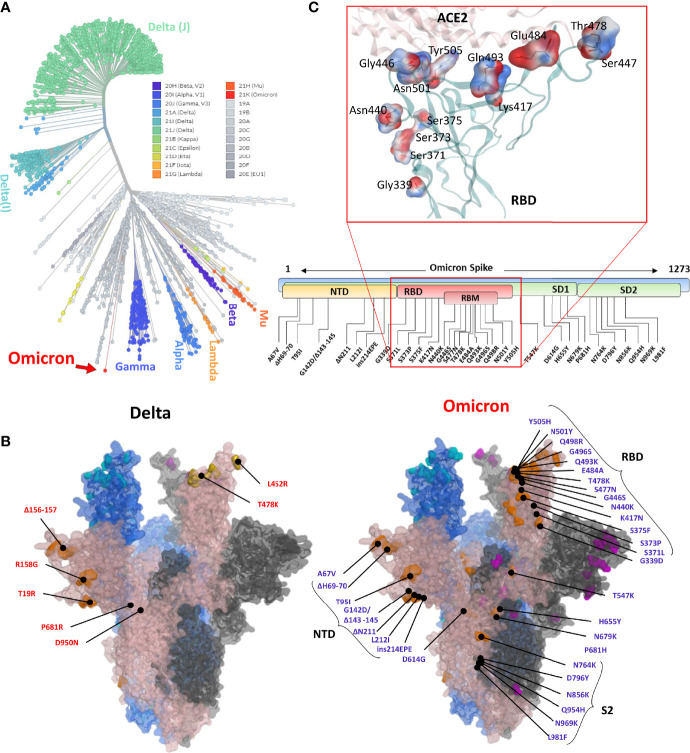
**(A)** Phylogeny of the Omicron and annotation of the mutation in Spike protein. The unrooted phylogenetic tree was constructed from the Nextstrain servers. Wuhan-Hu-1/2019 strains were taken as a reference sequence. **(B)** The full-length Delta and Omicron Spikes were built to annotate the relative (not exact) positions of the mutations on the surface map of Spike. **(C)** The amino acids mutated in the RBD of Omicron are shown concerning the ACE2 interface. Residues are colored according to the electrostatic map of the WT strain. The respective Omicron mutations are depicted in the panel below the RBD surface map.

Omicron is unique among the previously reported SARS-CoV-2 VOCs, showing multiple mutations in Spike and other genes. According to the unrooted phylogenic analysis using the global ~4,000 full-genome SARS-CoV-2 sequences from the Global Initiative on Sharing All Influenza Data (GISAID), Omicron stands distant from other VOCs (**Figure 1A**). A full-length trimeric 3D model was constructed by substituting the respective amino acids of previously reported reference (Wuhan strain, PDB ID: 7VNE) structure into Omicron. There are three deletion sites in the N-terminus domains (NTD) and at least 15 substitutions in the RBD region. Omicron Spike also harbors mutations that were reported in the previous VOCs such as K417, T478, E484, and N501 (**Figure 1B**). Of these, at least 11 mutations are involved in ACE2 binding (**Table 1**), substantially affecting their binding affinity (**Figure 1C**). In addition, Omicron Spike, compared with the prototype SARS-CoV-2, has three deletions, i.e., Δ69-70, Δ143-145, and Δ211, and one highly charged insertion, i.e., ins214EPE at 214 positions.

**Table 1:** RBD binding interface residues of ACE2 and therapeutic antibodies.

ACE2	CT-p59	Sotrovimab	Etesevimab	Bamlanivimab	AZD1061	AZD8895	Casirivimab	Imdevimab
**Lys417**	Arg403	Asn334	Arg403	Tyr449	Arg346	**Lys417**	**Lys417**	Arg346
Gly446	Tyr449	Leu335	**Lys417**	Gly482	Lys444	Ala475	Tyr453	Asn440
**Glu484**	Asn450	Glu340	Asp420	**Glu484**	Tyr449	Gly476	Ser477	Lys444
**Asn487**	Tyr453	Asn343	Tyr421	Gly485	Asn450	Ser477	**Glu484**	Tyr449
**Gln493**	**Glu484**	Thr345	Leu455	Phe486	**Glu484**	Thr478	Phe486	**Gln498**
Gly496	Phe486	Arg346	Asn460	**Gln493**		**Asn487**	Tyr489	
**Gln498**	**Gln493**	Lys356	Tyr473	Ser494		Tyr489	Leu492	
Thr500	Ser494		Ala475	Tyr495		**Gln493**		
Gly502	Tyr505		**Asn487**					
Tyr505			**Gln493**					

Bold residues are shared by ACE2 and mAbs on the RBD interface.

**Figure 2:**
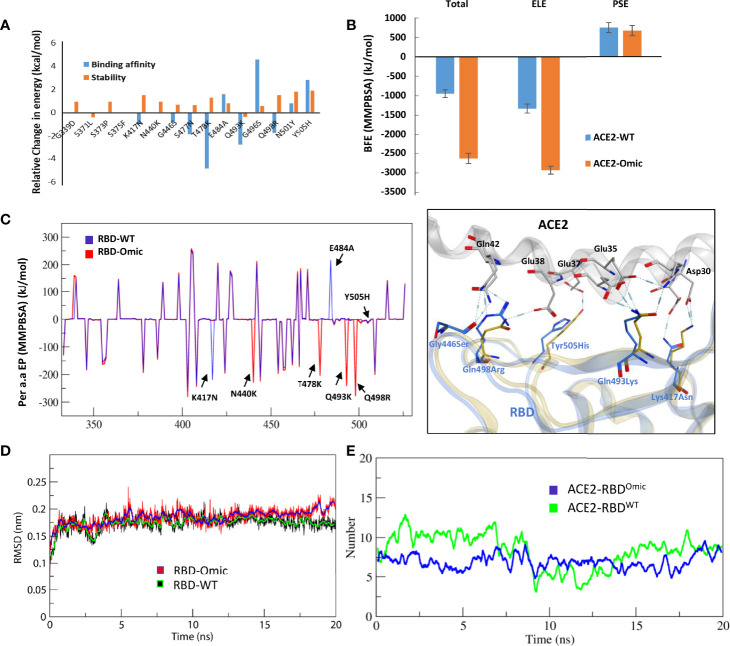
Relative effect of mutations in Omicron RBD on the ACE2 binding. **(A)** Effect of 15 individual mutations on the binding and stability of RBD^Omic^–ACE2 was monitored relative to that of RBD^WT^–ACE2. **(B)** The binding free energies (measured through MMPBSA) as consequences of all 15 mutations at once were monitored for both RBD^Omic^–ACE2 and RBD^WT^–ACE2. **(C)** Per-residue energy contribution was monitored, and the hotspots of RBD were labeled. The change in the hydrogen bond network of the selected hotspots is shown at the right. **(D, E)** Root mean square deviation and hydrogen bonds at the RBD–ACE2 interface as a function of time are displayed for both RBD^Omic^–ACE2 and RBD^WT^–ACE2 complexes.

We monitored the relative binding strength of RBD–ACE2 complexes of both prototype and Omicron strains using a protein design strategy and calculating binding affinity and stability changes in terms of relative change in energies. We observed that the individually substituted residues had a slight effect on the local stability of the RBD–ACE2 complexes (**Figure 2A**). However, by performing endpoint molecular mechanics generalized born surface area (MMGBSA) binding free energy calculation, we could demonstrate a substantial increase in the binding affinity by T478K, Q493K, and Q498R, leading to an overall increase in the binding affinity of the RBD^Omic^ with ACE2 (ΔG^WT^ = −64.65 kcal/mol < ΔG^Omic^ = −83.79 kcal/mol; **Supplementary Figure 1A**). We also investigated the change in electrostatic potential of the RBD^Omic^ relative to that of RBD^WT^ because the five residues in the RBM region of RBD are mutated from the polar to the positively charged residues (i.e., N440K, T478K, Q493K, Q498R, and Y505H). Surprisingly, we could observe that the electrostatic energy of ACE2–RBD^Omic^ was double as that of ACE2–RBD^WT^, which in turn doubled the polar solvation free energies of the ACE2–RBD^Omic^ (**Supplementary Figure 1A**). Per residues, energy distribution suggests that mutations in RBD^Omic^ directly participate in the binding, enhancing the binding strength of amino acids in the same network (**Supplementary Figure 1B**). To validate our finding, we performed molecular dynamics simulations (MDS) using GROMACS (11) and calculated their binding free energies using the widely acceptable and more authentic molecular mechanics Poisson–Boltzmann surface area (MMPBSA) approach (12). As expected, we observed that RBD^Omic^, compared with RBD^WT^, had over 2.5 (BFE = −2,642.5 kJ/mol) times stronger binding affinity toward ACE2 (BFE = −951.9 kJ/mol). In addition, the electrostatic potential of RBD^Omic^ was increased by ~1.5 times due to the polar-to-positive amino acids substitution (**Figure 2B**). In addition, energy perturbation per amino acid could confirm that the four amino acids, i.e., N440K, T478K, Q493K, and Q498R, directly contribute to the change of the total energy and the electrostatic potential, whereas K417N and E484A compensate the energy change (**Figure 2C**). Among 15 substituted amino acids, K417N and Y505H exhibited a slight reduction in binding energy due to the breakage of salt bridges between K417 of the RBD and D30 of ACE2; nonetheless, this breakage was compensated by the salt bridge between E35 of ACE2 and Q493K substitution in RBD^Omic^ (**Figure 2C**, right panel). Although the simulation time was short (20 ns), the root mean square deviation (RMSD) of the RBD proteins was not much different (**Figure 2D**). However, the number of hydrogen bonds between RBD^WT^–ACE2 showed a transient shift from high (*N* = ~7.5) to low (*N* = ~5) to high (*N* = ~7.5). This effect was not observed in RBD^Omic^–ACE2, and the hydrogen bond number remained consistent (*N* = ~7.5) (**Figure 2E**). Taken together, we suggest that Omicron binds ACE2 with greater affinity, partly explaining its increased transmissibility.

### Mutations in the RBD^Omic^ Deteriorate the Binding of Therapeutic Antibodies and Garble Their Epitopes on RBD

**Figure 3:**
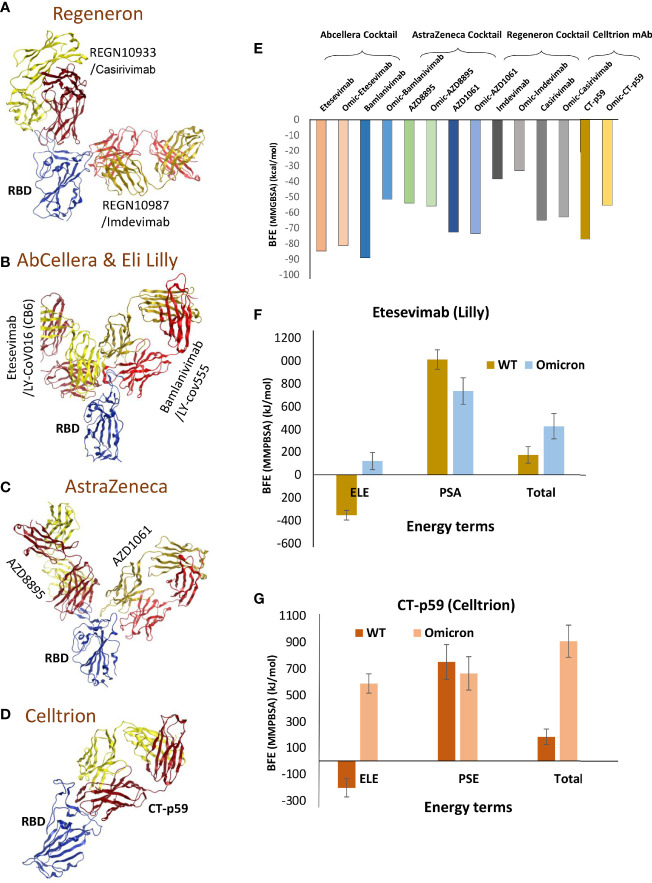
Mutations in the Omicron RBD distort the epitopes of therapeutic mAbs. **(A–D)** Crude epitopes of seven selected mAbs are shown on the RBD. Antibodies used as cocktails are labeled with their sponsors. All variable light chains are colored yellow or orange and variable heavy chains are colored red. **(E)** Changes in the binding affinity of the RBD^Omic^–mAbs relative to RBD^WT^–mAbs are shown. The binding energies were calculated through endpoint MMGBSA. **(F)** The binding free energies (measured through MMPBSA) as consequences of all 15 mutations at once were monitored for RBD^Omic^–etesevimab and RBD^WT^–etesevimab. **(G)** The binding free energies (measured through MMPBSA) as consequences of all 15 mutations at once were monitored for RBD^Omic^–CT-p59 and RBD^WT^–CT-p59.

To evaluate whether the mutations that strengthen the RBD^Omic^–ACE2 interaction affect the RBD-targeting COVID-19 therapeutic antibodies, we constructed structural models of eight monoclonal antibodies (mAbs) bound to RBD^Omic^. The antibodies like CT-p59, developed by Celltrion from the peripheral blood mononuclear cells (PBMCs) derived from a convalescent plasma of COVID-19 patients, and sotrovimab are used as solo COVID-19 therapeutics undergoing phase III clinical trials (13, 14). The other six mAbs were approved for COVID-19 therapies on an emergency basis (15), which are used as cocktail therapy to tackle the immune escape by the newly acquired mutants (**Figures 3A–D**).

Since mAbs in their respective cocktail therapy regimen do not share overlapping epitopes on the RBD, except etesevimab and bamlanivimab (sponsored by AbCellera) where the light chain variable domains show a slight clash (**Figure 3B** and **Table 1**) and are capable of neutralizing the virus independently, we investigated the change in their interface and binding strength of the mAbs with RBD^Omic^ individually. Remarkably, we found a substantial drop in the total binding energies of bamlanivimab and CT-p59 when bound to RBD^Omic^ (**Figure 3E**). The total binding energy is the sum of the four energies (listed in **Table 2**). We could see that vdW (Van der Waals potentials) and SA (solvation free energy) energies did not affect the binding strength; however, electrostatic potentials (ELE) had a significant shift in the calculated energies. The magnitude of the change in ELE energies was similar to that of RBD^Omic^–ACE2; however, the effect was opposite, i.e., in RBD^Omic^–ACE2 ELE energies favor the binding, whereas RBD^Omic^–mAbs ELE opposed the binding strength (**Supplementary Figure 1C**). All the mAbs showed a significant drop in ELE energies, but AZD1061 (AstraZeneca) showed a slight drop (RBD-AZD1061 = −204.24 kcal/mol > RBD^Omic^-AZD1061 = −112.35 kcal/mol). To validate the relative change of total binding energies and the shift of the electrostatic potential of RBD^Omic^–mAbs, we analyzed the MDS of the two complexes (i.e., RBD^Omic^–etesevimab and RBD^Omic^–CT-p59) and calculated their BFE using MMPBSA. Interestingly, we found that the change of the electrostatic potential of both complexes exhibited a similar trend for both MMGBSA and MMPBSA approaches. The MMPBSA values were calculated as the statistical outcome of 100 frames extracted from the 20-ns MDS trajectory (**Figures 3F, G**). The total binding energies for RBD^Omic^–mAbs complexes were substantially higher compared with the RBD^WT^–mAbs, suggesting that Omicron can escape both etesevimab and CT-p59 (regdanvimab) (**Figures 3F, G**).

**Table 2:** The binding energies of RBD^Omic^ and RBD^WT^ with seven therapeutic mAbs are listed.

Sponsor	mAbs	VDW	ELE	GB	SA	Total
AbCellera and Eli Lilly	Etesevimab	−131.41	−179.48	243.28	−17.15	−84.76
	Omic-Ete	−147.42	65.04	19.31	−18.16	−81.24
	Bamlanivimab	−95.81	−13.97	33.7	−13.01	−89.1
	Omic-Bam	−108.74	602.59	−532.27	−13.12	−51.54
AstraZeneca	AZD8895	−84.37	−30.35	71.27	−10.46	−53.92
	Omic-Az95	−96.77	76.73	−24.25	−11.62	−55.91
	AZD1061	−94.96	−204.24	238.04	−11.49	−72.64
	Omic-Az61	−98.83	−112.35	149.92	−12.29	−73.55
Regeneron	Imdevimab	−76.1	−27.36	74.73	−9.67	−38.4
	Omic-Imd	−81.32	−5.84	64.23	−10.15	−33.08
	Casirivimab	−103.52	−174.81	228.04	−14.66	−64.94
	Omic-Cas	−113.25	−55.14	119.9	−14.42	−62.91
Celltrion	CT-p59	−105.66	−7.38	49.61	−13.75	−77.18
	Omic-CT-p59	−114.85	352.19	−278.48	−14.27	−55.4

mAbs, monoclonal antibodies; VDW, Van der Waals potentials; ELE, electrostatic potentials; GB, polar solvation potentials (generalized born model); SA, non-polar contribution to the solvation free energy calculated by an empirical model.

**Figure 4:**
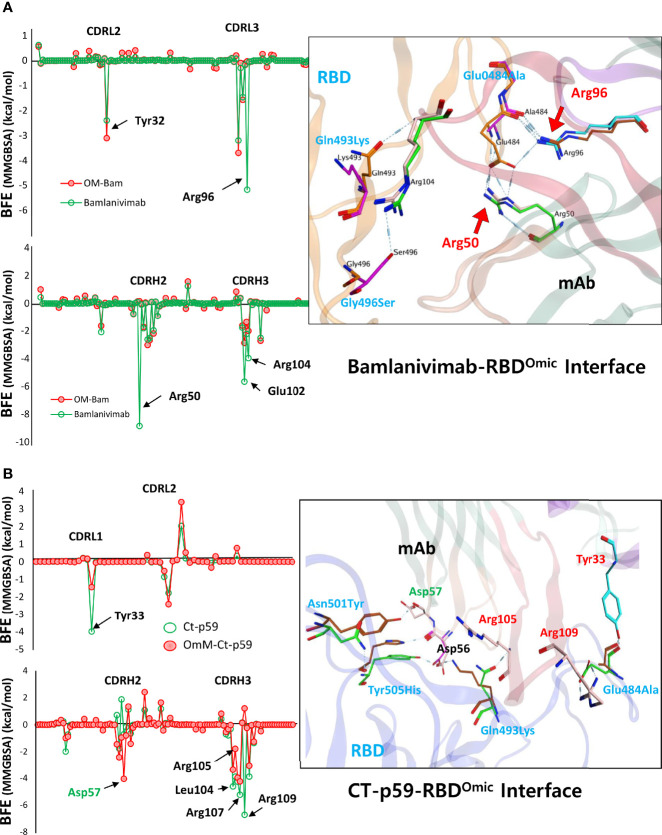
Per-residue changes in the binding affinity of RBD–mAbs were monitored and the hotspots on CDRs of **(A)** bamlanivimab and **(B)** CT-p59 are labeled. The change in the hydrogen bond network of the selected hotspots is shown at the right.

Next, to evaluate which mutations are mainly involved in weakening the RBD^Omic^–mAbs interactions, we calculated per amino acid energy perturbation for CT-p59 and bamlanivimab when bound to RBD^WT^ and RBD^Omic^. We observed that two hotspots, i.e., R96 in CDRL3 and R50 in CDRH2 of bamlanivimab, established highly stable salt bridges with the E484 of RBD^WT^, losing their binding entirely upon E484A mutation in RBD^Omic^ (**Figure 4A**). In addition, E102 and R104 in CDRH3 showed a 50% reduction in binding energies. Similarly, the hotspots in CDRL1 and CDRH3 lost their bindings due to the mutations of E484A, Q493K, and Y505H in RBD^Omic^. By contrast, N501Y slightly strengthens the binding, establishing a hydrogen bond with D57 in CDRH2 (**Figure 4B**). Next, we extended our search and used two more SARS-CoV-2 neutralizing antibodies, C102 and C105, isolated from the convalescent plasma of a single donor for their RBD^Omic^ affinity (16). Both mAbs bind to an overlapping epitope on the RBM and predominantly utilize the heavy chain variable domain (VH) CDRs as main paratopes (**Supplementary Figure 2A**). Like other RBD-binding mAbs, both C102 and C105 exhibited a drastic reduction in binding affinity driven by a significant change in the electrostatic potential (**Supplementary Figures 2B, C**). To investigate how sotrovimab retains its neutralization efficacy, we constructed the RBD^Omic^–sotrovimab model and found that sotrovimab binds to a highly conserved epitope on the RBD and, among 15 mutations in the RBD^Omic^, faces only G339D mutation. The retained Omicron neutralization in pseudovirus assay (17) may indicate that the salt bridges between the CDRH3 and RBD may override the clash between RBD^Omic^ D339 and Y100 in CDRH3 that could potently destabilize the RBD^Omic^–sotrovimab interaction (**Supplementary Figure 2D**). These results suggest that mutations in the Omicron Spike are precisely designed by the virus, facilitating receptor binding but hindering antibody binding simultaneously and that antibodies recognizing conserved epitope on the Spike of SARS-CoV-2 variants could be used as pan-variant therapeutics. Overall, the escape of Omicron from a large pool of antibodies, especially those approved by the FDA after undergoing extensive clinical trials and safety measures, raises serious concerns about the efficacy of therapeutic mAbs in Omicron-infected patients.

## Discussion

To this end, it is well known that SARS-CoV-2 is rapidly evolving and makes at least two mutations per month in its genome (18, 19). The virus is capable of adapting to the host environment by increasing transmissibility and evading immune response, as exemplified by the continuous rise of VOCs (20, 21). Although tremendous efforts have been made in vaccine development and COVID-19 therapeutics, including mAbs and COVID-19 pills by Merck, the emergence of VOCs has raised concerns over the efficacy of neutralizing antibodies (21, 22). Even though these variants had a limited number of mutations, they successfully escaped the immune response, at least partly if not entirely. Omicron harbors four or five times more mutations in the Spike protein than other SARS-CoV-2 VOCs and raise more serious concerns (see **Figure 1B**).

We used the previously available structural data of Spike RBD-binding antibodies, Spike itself, and Spike–ACE2 complexes and constructed the mutant Omicron Spike. Omicron Spike contains some of the mutations reported in the previous VOCs. In particular, D614 enhances the receptor binding by increasing its “up” conformation and the overall density of Spike protein at the surface of the virus (23, 24). In addition, five of the amino acids within the RBD region are mutated from polar to positively charged residues (K, R, or H) that paradoxically enhance the receptor binding and weaken the Spike neutralizing interactions (**Figures 2C** and **4**). The RBD mutation has been mapped to predict the neutralization escape from REGN-COV2 (a cocktail of REGN10933 and REGN10987) and LY-CoV016. Such mutations have already been found in patients with persistently infected COVID-19 since late 2020 or early 2021 (25). Single E406W mutation can lead to the viral escape from both antibodies in REGN-COV2, whereas F486K has been reported to escape REGN10933. N440K and K444Q can escape against REGN10987, while K417N, N460T, and A475V can successfully escape against LY-CoV016 antibody (AbCellera), currently approved by the FDA for COVID-19 therapy (25). Unfortunately, Omicron has mutations or amino acids adjacent to those predicted to escape the neutralization of antibodies.

Among the investigated antibodies here, we suggest that AZD1061 may be able to retain Omicron neutralization (**Figure 3E**). During our study, two research groups investigated the neutralization escape of Omicron from the same set of antibodies. They reported consistent results for the mAbs sponsored by Regeneron and AbCellera. Nonetheless, they demonstrated that the Omicron pseudovirus neutralization findings differed in AZD1061 (cilgavimab) and AZD8895 (tixagevimab). In support of our results, Planas et al. have shown that AZD1061 (cilgavimab) and the cocktail (AZD1061+AZD8895), but not AZD8895 alone, could retain the Omicron pseudovirus neutralization (17).

On the other hand, Cao et al. have demonstrated that AZD8895, but not AZD1061, binds Omicron with lower affinity and slightly neutralizes the Omicron pseudovirus at a very higher concentration (26). However, they did not examine the effect of the AZD1061+AZD8895 cocktail on the Omicron. The above studies suggested that sotrovimab (VIR-7831/GSK4182136/S309) holds the promising neutralization efficacy against the Omicron pseudovirus. Thus, we constructed an RBD^Omic^–sotrovimab model to investigate how sotrovimab retains its neutralization efficacy. Sotrovimab could bind to the highly conserved epitope on the RBD, and among the 15 mutations in the RBD^Omic^, it faces only G339D mutation.

Although the pathological manifestations are thus far reported to be mild, the threat of Omicron is global, and it is also quite clear that the new variant is more transmissible than Delta. Vaccination has been reported to significantly drop the COVID-19 infection of emerging VOCs, including Delta (27). However, the sera from convalescent subjects infected with different variants of SARS-CoV-2 including Alpha, Beta, Gamma, and Delta and vaccines were found to be ineffective against Omicron. Nonetheless, immunity boosted by the third dose of vaccines or vaccinees infected by the Delta strain has shown effect against Omicron (28). Another study has also reported some preliminary data about the ineffectiveness of vaccines against Omicron. The sera from individuals with the 5-month post-vaccination with Pfizer (29) or AstraZeneca vaccine have failed to inhibit Omicron (30, 31). Similarly, the sera from 6- to 12-month post-infection individuals could not neutralize the new variant. Nevertheless, 5- to 31-fold lower neutralization of Omicron compared with Delta has been reported by boosters and the previously infected vaccines (17). Thus, Omicron can escape against the therapeutic and vaccine-elicited antibodies. Our study and the preliminary data from other studies consistently suggest that a cocktail of Evusheld (AstraZeneca mAbs) and sotrovimab (GSK, S203 mAb) could effectively neutralize the Omicron.

## Methods

### Model Construction and Optimization

For the full-length trimeric Spike, a previously reported PDB ID: 7VNE was used to rebuild the Omicron Spike protein using a Swiss-Model server (32). Other structures used in this study are listed as follows: RBD–ACE2 (PDB ID: 6MOJ), RBD–etesevimab (PDB ID: 7C01), RBD–bamlanivimab (PDB ID: 7KMG), RBD–CT-p59 (PDB ID: 7CM4), RBD–AZD1061 (PDB ID: 7I7E), RBD–AZD8895 (PDB ID: 7I7E), RBD–casirivimab (PDB ID: 6XDG), RBD–imdevimab (PDB ID: 6XDG), and RBD–sotrovimab (PDB ID: 7R6X). For constructing the mutant RBD, free BIOVIA Discovery Studio Visualizer was used (http://www.accelrys.com). All complexes were solvated with TIP3P water cubic box of dimension boundaries extended to 10 Å from protein atoms and neutralized with counter ions, Na^+^/Cl^−^, wherever needed. The neutralized systems were energy minimized in GROMACS 2019.6 (33) using CHARMM37 force field (34) and steep descent algorithm. For endpoint binding free energy calculations, the HawkDock server was utilized (35).

### Molecular Dynamics Simulation

To calculate the binding free energies of RBD^Omic^ with ACE2 and antibodies, we utilized the GROMACS package for the generation of trajectories and the MMPBSA tool for the free energy perturbation. Each system was solvated in a dodecahedron box filled with TIP3P water model and neutralized by adding counter ions (Na^+^/Cl^−^). The neutralized systems were energy minimized as stated above. Next, a two-step equilibration was set up under constant temperature (NVT) and constant pressure (NPT) of 0.2 ns, and the systems were equilibrated. The temperature and pressure were coupled with v-rescale (modified Berendsen thermostat) and Berendsen, respectively (36). The long-range electrostatic interactions were computed by utilizing the particle mesh Ewald algorithm (37). Each system was simulated for 20 ns with all constrains removed. For the calculation of RMSD and hbonds, MD trajectories were converted by removing the jumps and translational and rotational motions using −pbc nojump and −fit rot+trans flags under the trjconv tool in GROMACS. For MMPBSA, every 20th frame was extracted from a 20-ns trajectory in a separate trajectory.

### Binding Free Energy Calculation

The MMPBSA (12) approach is best suited for calculating binding free energies of the ligands bound to the same target. Here, RBD is the main target, whereas mAbs and ACE2 are considered as ligands. GROMACS is equipped with g_mmpbsa tool which was used for the calculation of binding free energies. The topology of each system was generated through the older version of GROMACS (v 5.0) as the MMPBSA package has not been updated by the developer till now. The binding energies were calculated according to the equations described in our previous study (38).

### Computational Tools Used in This Project

For protein structure visualization, VMD (39), PyMOL (https://pymol.org), and Chimaera Chimera (40) packages were used. For electrostatic surfaces, isolation of the proteins, APBS and APBSrun plugins in PyMOL and VMD were utilized. The interfaces of RBD^WT^ and RBD^Omic^ with mAbs and ACE2 were analyzed by the online server PDBePISA (v1.52) (41), and the binding contribution of individual amino acids was determined. For endpoint binding free energy calculations, the HawkDock server was utilized (35). The hotspot results were validated through the DrugScorePPI web server (42). The unrooted phylogenetic tree was constructed from the Nextstrain (43) servers using ~4,000 full-length SARS-CoV-2 sequences from the GISAID (44) database with reference to Wuhan-Hu-1/2019 as a reference sequence.

## Data Availability Statement

The raw data supporting the conclusions of this article will be made available by the authors, without undue reservation.

## Author Contributions

MS and HW contributed toward conceptualization of the project and designed the methodology. MS and HW wrote the original manuscript draft. HW supervised the study and provided funding acquisition. All authors contributed to the article and approved the submitted version.

## Funding

This research was supported by grants from the National Research Foundation of Korea (NRF) funded by the Ministry of Science and ICT (MSIT) (NRF-2017M3C9A6047620, NRF-2019R1A5A2026045, and NRF-2017M3A9B6061509) and grant from the Korea Health Industry Development Institute (KHIDI) funded by the Ministry of Health & Welfare, Republic of Korea (HI21C1003). In addition, this study was also supported by KREONET (Korea Research Environment Open NETwork), which is managed and operated by KISTI (Korea Institute of Science and Technology Information).

## Conflict of Interest

The authors declare that the research was conducted in the absence of any commercial or financial relationships that could be construed as a potential conflict of interest.

## Publisher’s Note

All claims expressed in this article are solely those of the authors and do not necessarily represent those of their affiliated organizations, or those of the publisher, the editors and the reviewers. Any product that may be evaluated in this article, or claim that may be made by its manufacturer, is not guaranteed or endorsed by the publisher.

## References

[1] Garcia-Beltran WF Lam EC St Denis K Nitido AD Garcia ZH Hauser BM. Multiple SARS-CoV-2 Variants Escape Neutralization by Vaccine-Induced Humoral Immunity. Cell (2021) 184(9):2523. doi: 10.1016/j.cell.2021.04.006 33930298 PMC8082941

[2] Mlcochova P Kemp SA Dhar MS Papa G Meng B Ferreira I. SARS-CoV-2 B.1.617.2 Delta Variant Replication and Immune Evasion. Nature (2021) 599(7883):114–9. doi: 10.1038/s41586-021-03944-y PMC8566220 34488225

[3] Yi C Sun X Lin Y Gu C Ding L Lu X. Comprehensive Mapping of Binding Hot Spots of SARS-CoV-2 RBD-Specific Neutralizing Antibodies for Tracking Immune Escape Variants. Genome Med (2021) 13(1):164. doi: 10.1186/s13073-021-00985-w 34649620 PMC8515915

[4] Planas D Veyer D Baidaliuk A Staropoli I Guivel-Benhassine F Rajah MM. Reduced Sensitivity of SARS-CoV-2 Variant Delta to Antibody Neutralization. Nature (2021) 596(7871):276–80. doi: 10.1038/s41586-021-03777-9 34237773

[5] Callaway E. Heavily Mutated Omicron Variant Puts Scientists on Alert. Nature (2021). doi: 10.1038/d41586-021-03552-w 34824381

[6] Shah M Woo HG. Molecular Perspectives of SARS-CoV-2: Pathology, Immune Evasion, and Therapeutic Interventions. Mol Cells (2021) 44(6):408–21. doi: 10.14348/molcells.2021.0026 PMC8245319 34059561

[7] Meng B Ferreira IATM Abdullahi A Saito A Kimura I Yamasoba D. SARS-CoV-2 Omicron Spike Mediated Immune Escape, Infectivity and Cell-Cell Fusion. bioRxiv [Preprint] (2021). doi: 10.1101/2021.12.17.473248

[8] Mu J Fang Y Yang Q Shu T Wang A Huang M. SARS-CoV-2 N Protein Antagonizes Type I Interferon Signaling by Suppressing Phosphorylation and Nuclear Translocation of STAT1 and STAT2. Cell Discov (2020) 6:65. doi: 10.1038/s41421-020-00208-3 32953130 PMC7490572

[9] Dobano C Santano R Jimenez A Vidal M Chi J Rodrigo Melero N. Immunogenicity and Crossreactivity of Antibodies to the Nucleocapsid Protein of SARS-CoV-2: Utility and Limitations in Seroprevalence and Immunity Studies. Transl Res (2021) 232:60–74. doi: 10.1016/j.trsl.2021.02.006 33582244 PMC7879156

[10] Helmsdal G Hansen OK Møller LF Christiansen DH Petersen MS Kristiansen MF. Omicron Outbreak at a Private Gathering in the Faroe Islands, Infecting 21 of 33 Triple-Vaccinated Healthcare Workers. medRxiv (2021). doi: 10.1101/2021.12.22.21268021 PMC9383377 35134167

[11] Pronk S Pall S Schulz R Larsson P Bjelkmar P Apostolov R. GROMACS 4.5: A High-Throughput and Highly Parallel Open Source Molecular Simulation Toolkit. Bioinformatics (2013) 29(7):845–54. doi: 10.1093/bioinformatics/btt055 PMC3605599 23407358

[12] Kumari R Kumar R. Open Source Drug Discovery C, Lynn A. G_Mmpbsa–a GROMACS Tool for High-Throughput MM-PBSA Calculations. J Chem Inf Model (2014) 54(7):1951–62. doi: 10.1021/ci500020m 24850022

[13] Kim JY Jang YR Hong JH Jung JG Park JH Streinu-Cercel A. Safety, Virologic Efficacy, and Pharmacokinetics of CT-P59, a Neutralizing Monoclonal Antibody Against SARS-CoV-2 Spike Receptor-Binding Protein: Two Randomized, Placebo-Controlled, Phase I Studies in Healthy Individuals and Patients With Mild SARS-CoV-2 Infection. Clin Ther (2021) 43(10):1706–27. doi: 10.1016/j.clinthera.2021.08.009 PMC8380488 34551869

[14] Gupta A Gonzalez-Rojas Y Juarez E Crespo Casal M Moya J Falci DR. Early Treatment for Covid-19 With SARS-CoV-2 Neutralizing Antibody Sotrovimab. N Engl J Med (2021) 385(21):1941–50. doi: 10.1056/NEJMoa2107934 34706189

[15] Taylor PC Adams AC Hufford MM de la Torre I Winthrop K Gottlieb RL. Neutralizing Monoclonal Antibodies for Treatment of COVID-19. Nat Rev Immunol (2021) 21(6):382–93. doi: 10.1038/s41577-021-00542-x PMC8054133 33875867

[16] Chen EC Gilchuk P Zost SJ Suryadevara N Winkler ES Cabel CR. Convergent Antibody Responses to the SARS-CoV-2 Spike Protein in Convalescent and Vaccinated Individuals. Cell Rep (2021) 36(8):109604. doi: 10.1016/j.celrep.2021.109604 34411541 PMC8352653

[17] Planas D Saunders N Maes P Guivel-Benhassine F Planchais C Buchrieser J. Considerable Escape of SARS-CoV-2 Variant Omicron to Antibody Neutralization. bioRxiv [Preprint] (2021). doi: 10.1101/2021.12.14.472630 35016199

[18] Duchene S Featherstone L Haritopoulou-Sinanidou M Rambaut A Lemey P Baele G. Temporal Signal and the Phylodynamic Threshold of SARS-CoV-2. Virus Evol (2020) 6(2):veaa061. doi: 10.1093/ve/veaa061 33235813 PMC7454936

[19] Worobey M Pekar J Larsen BB Nelson MI Hill V Joy JB. The Emergence of SARS-CoV-2 in Europe and North America. Science (2020) 370(6516):564–70. doi: 10.1126/science.abc8169 PMC7810038 32912998

[20] Harvey WT Carabelli AM Jackson B Gupta RK Thomson EC Harrison EM. SARS-CoV-2 Variants, Spike Mutations and Immune Escape. Nat Rev Microbiol (2021) 19(7):409–24. doi: 10.1038/s41579-021-00573-0 PMC8167834 34075212

[21] Krause PR Fleming TR Longini IM Peto R Briand S Heymann DL. SARS-CoV-2 Variants and Vaccines. N Engl J Med (2021) 385(2):179–86. doi: 10.1056/NEJMsr2105280 PMC8262623 34161052

[22] Kemp SA Collier DA Datir RP. SARS-CoV-2 Evolution During Treatment of Chronic Infection. Nature (2021) 592:277–82. doi: 10.1038/s41586-021-03291-y PMC7610568 33545711

[23] Mansbach RA Chakraborty S Nguyen K Montefiori D Korber B Gnanakaran S. The SARS-CoV-2 Spike Variant D614G Favors an Open Conformational State. Sci Adv (2020) 7(16):eabf3671. doi: 10.1126/sciadv.abf3671 PMC8051874 33863729

[24] Zhang L Jackson CB Mou H Ojha A Rangarajan ES Izard T. SARS-CoV-2 Spike-Protein D614G Mutation Increases Virion Spike Density and Infectivity. Nat Commun (2020) 11(1):6013. doi: 10.1038/s41467-020-19808-4 33243994 PMC7693302

[25] Starr TN Greaney AJ Addetia A Hannon WW Choudhary MC Dingens AS. Prospective Mapping of Viral Mutations That Escape Antibodies Used to Treat COVID-19. Science (2021) 371(6531):850–4. doi: 10.1126/science.abf9302 PMC7963219 33495308

[26] Cao Y Wang J Jian F Xiao T Song W Yisimayi A. Omicron Escapes the Majority of Existing SARS-CoV-2 Neutralizing Antibodies. bioRxiv [Preprint] (2021). doi: 10.1101/2021.12.07.470392 PMC8866119 35016194

[27] Tartof SY Slezak JM Fischer H Hong V Ackerson BK Ranasinghe ON. Effectiveness of mRNA BNT162b2 COVID-19 Vaccine Up to 6 Months in a Large Integrated Health System in the USA: A Retrospective Cohort Study. Lancet (2021) 398(10309):1407–16. doi: 10.1016/S0140-6736(21)02183-8 PMC8489881 34619098

[28] Dejnirattisai W Huo J Zhou D Zahradník J Supasa P Liu C. Omicron-B.1.1.529 Leads to Widespread Escape From Neutralizing Antibody Responses. bioRxiv [Preprint] (2021). doi: 10.1101/2021.12.03.471045 PMC8723827 35081335

[29] Cele S Jackson L Khoury DS Khan K Moyo-Gwete T Tegally H. SARS-CoV-2 Omicron has Extensive But Incomplete Escape of Pfizer BNT162b2 Elicited Neutralization and Requires ACE2 for Infection. medRxiv [Preprint] (2021). doi: 10.1101/2021.12.08.21267417

[30] Wilhelm A Widera M Grikscheit K Toptan T Schenk B Pallas C. Reduced Neutralization of SARS-CoV-2 Omicron Variant by Vaccine Sera and Monoclonal Antibodies. medRxiv [Preprint] (2021). doi: 10.1101/2021.12.07.21267432

[31] Rössler A Riepler L Bante D Laer DV Kimpel J. SARS-CoV-2 B.1.1.529 Variant (Omicron) Evades Neutralization by Sera From Vaccinated and Convalescent Individuals. medRxiv [Preprint] (2021). doi: 10.1101/2021.12.08.21267491

[32] Waterhouse A Bertoni M Bienert S Studer G Tauriello G Gumienny R. SWISS-MODEL: Homology Modelling of Protein Structures and Complexes. Nucleic Acids Res (2018) 46(W1):W296–303. doi: 10.1093/nar/gky427 PMC6030848 29788355

[33] Abraham MJ Murtola T Schulz R Páll S Smith JC Hess B. GROMACS: High Performance Molecular Simulations Through Multi-Level Parallelism From Laptops to Supercomputers. SoftwareX (2015) 1–2:19–25. doi: 10.1016/j.softx.2015.06.001

[34] Huang J Rauscher S Nawrocki G Ran T Feig M de Groot BL. CHARMM36m: An Improved Force Field for Folded and Intrinsically Disordered Proteins. Nat Methods (2017) 14(1):71–3. doi: 10.1038/nmeth.4067 PMC5199616 27819658

[35] Feng T Chen F Kang Y Sun H Liu H Li D. HawkRank: A New Scoring Function for Protein-Protein Docking Based on Weighted Energy Terms. J Cheminform (2017) 9(1):66. doi: 10.1186/s13321-017-0254-7 29282565 PMC5745212

[36] Bussi G Donadio D Parrinello M. Canonical Sampling Through Velocity Rescaling. J Chem Phys (2007) 126(1):014101. doi: 10.1063/1.2408420 17212484

[37] Wang H Dommert F Holm C. Optimizing Working Parameters of the Smooth Particle Mesh Ewald Algorithm in Terms of Accuracy and Efficiency. J Chem Phys (2010) 133(3):034117. doi: 10.1063/1.3446812 20649318

[38] Shah M Ahmad B Choi S Woo HG. Mutations in the SARS-CoV-2 Spike RBD are Responsible for Stronger ACE2 Binding and Poor Anti-SARS-CoV Mabs Cross-Neutralization. Comput Struct Biotechnol J (2020) 18:3402–14. doi: 10.1016/j.csbj.2020.11.002 PMC7657873 33200028

[39] Humphrey W Dalke A Schulten K. VMD: Visual Molecular Dynamics. J Mol Graphics (1996) 14(1):33–8. doi: 10.1016/0263-7855(96)00018-5 8744570

[40] Pettersen EF Goddard TD Huang CC Couch GS Greenblatt DM Meng EC. UCSF Chimera–a Visualization System for Exploratory Research and Analysis. J Comput Chem (2004) 25(13):1605–12. doi: 10.1002/jcc.20084 15264254

[41] Krissinel E Henrick K. Inference of Macromolecular Assemblies From Crystalline State. J Mol Biol (2007) 372(3):774–97. doi: 10.1016/j.jmb.2007.05.022 17681537

[42] Kruger DM Gohlke H. DrugScorePPI Webserver: Fast and Accurate in Silico Alanine Scanning for Scoring Protein-Protein Interactions. Nucleic Acids Res (2010) 38(Web Server issue):W480–6. doi: 10.1093/nar/gkq471 PMC2896140 20511591

[43] Hadfield J Megill C Bell SM Huddleston J Potter B Callender C. Nextstrain: Real-Time Tracking of Pathogen Evolution. Bioinformatics (2018) 34(23):4121–3. doi: 10.1093/bioinformatics/bty407 PMC6247931 29790939

[44] Shu Y McCauley J. GISAID: Global Initiative on Sharing All Influenza Data - From Vision to Reality. Euro Surveill (2017) 22(13):30494. doi: 10.2807/1560-7917.ES.2017.22.13.30494 28382917 PMC5388101

[45] Shah M Woo HG. Omicron: A Heavily Mutated SARS-CoV-2 Variant Exhibits Stronger Binding to ACE2 and Potently Escape Approved COVID-19 Therapeutic Antibodies. bioRxiv [Preprint] (2021). doi: 2021:2021.12.04.471200 10.3389/fimmu.2021.830527 PMC8819067 35140714

