# Supplementary material for: Omicron: A Heavily Mutated SARS-CoV-2 Variant Exhibits Stronger Binding to ACE2 and Potently Escapes Approved COVID-19 Therapeutic Antibodies
Source: Front Immunol. 2022 Jan 24;12:830527. doi: 10.3389/fimmu.2021.830527 (PMC8819067; doi:10.3389/fimmu.2021.830527)
Supplement: Supplementary file 1 [file DataSheet_1.docx]

# **Omicron: A heavily mutated SARS-CoV-2 variant exhibits stronger binding to ACE2 and potently escape approved COVID-19 therapeutic antibodies**

Masaud Shah*^1^* and Hyun Goo Woo*^1,2,^**

*^1^*Department of Physiology, Ajou University School of Medicine, Suwon, Republic of Korea

*^2^Department of Biomedical Science, Graduate School, Ajou University, Suwon, Korea*

*Corresponding author

Hyun Goo Woo, M.D., Ph.D.

Tel: 82-31-219-5045,

Fax number: 82-31-219-5049,

E-mail address: [hg@ajou.ac.kr](mailto:hg@ajou.ac.kr)

# **Supplementary data**

**Supplementary Figure 1**

**
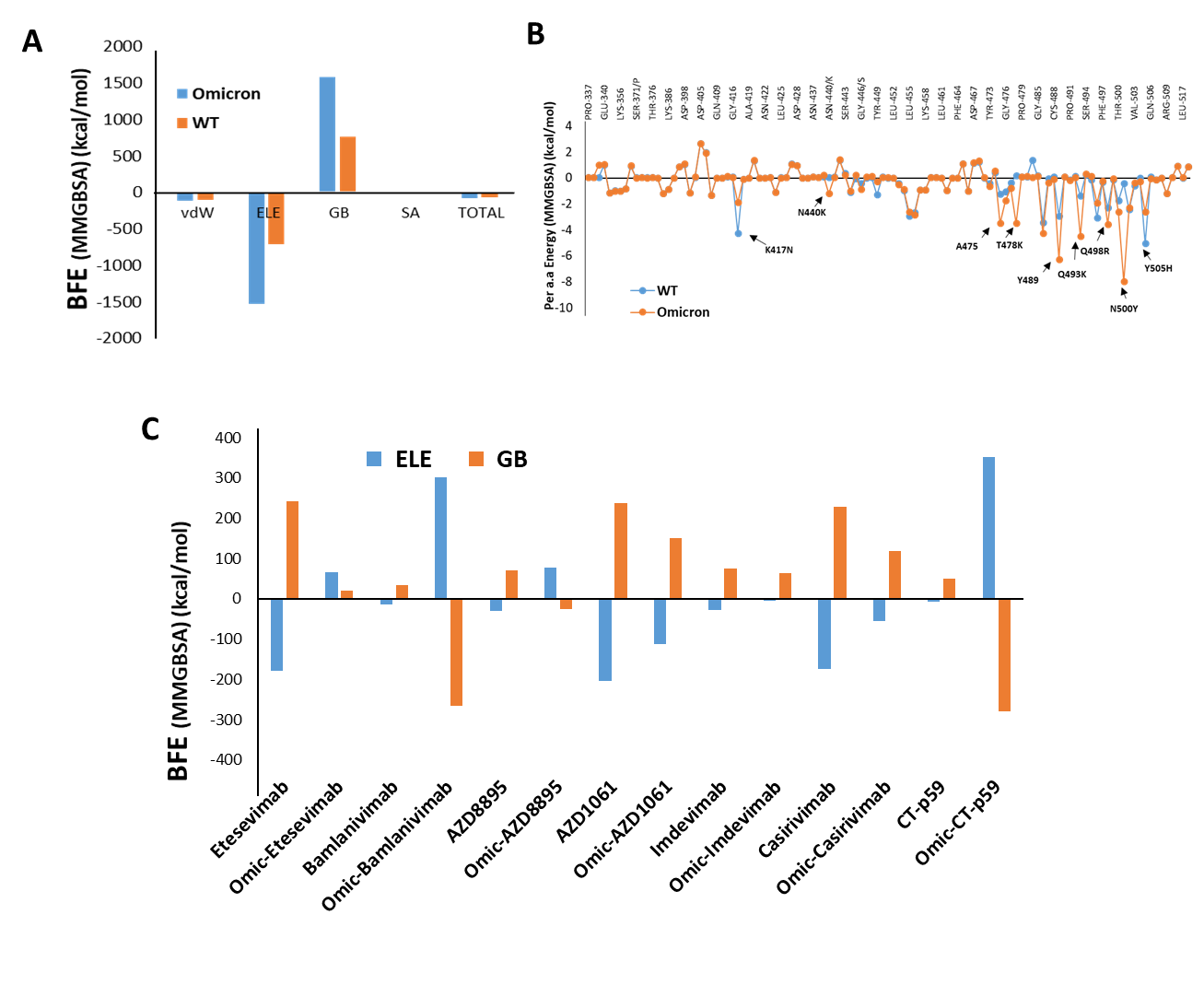
 Supplementary Figure 1. Change in the binding affinities of RBD^Omic^ with ACE2 and antibodies**. **A**) The overall binding energies (measured through endpoint MM/GBSA) as consequences of all 15 mutations at once were monitored for both RBD^Omic^‑ACE2 and RBD^WT^‑ACE2. **B**) Per-residue change in the binding affinity was monitored, and the hotspots of RBD were labeled. The change in the hydrogen bonds network of the elected hotspots is shown at the right. **C**) Changes in the binding affinity of the RBD^Omic^-mAbs relative to RBD^WT^-mAbs are calculated using MM/GBSA changes in the electrostatic potentials and polar solvation energies are shown for each RBD-mAb complex.

**
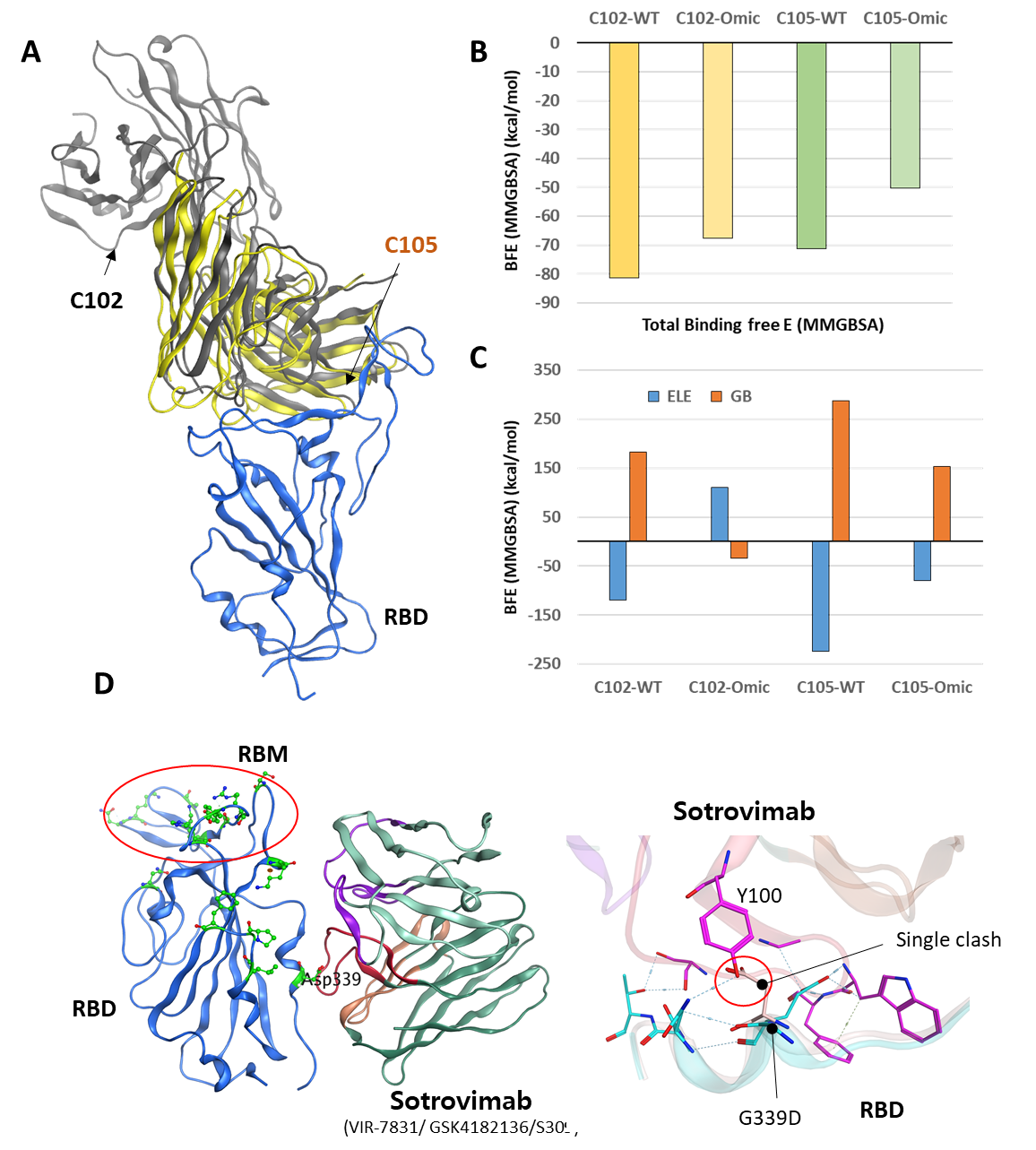
**

**Supplementary Figure 2. The epitopes and change in the binding affinities of RBD binding antibodies.** **A**) Crude epitopes of C102 and C105 antibodies selected from convalescent plasma are shown on the RBD. **B**) Total binding energies (measured through endpoint MM/GBSA) as consequences of all 15 mutations at once were monitored for both RBD^Omic^‑C102 and RBD^Omic^‑C105 relative to RBD^WT^. **C**) The epitope of Sotrovimab is shown on the RBD^Omic^. Sotrovimab binds a conserved epitope, away from the densely mutated RBM.
